# Rapid progression of cardiovascular-kidney-metabolic syndrome drives accelerated frailty trajectories: A longitudinal cohort study

**DOI:** 10.1097/MD.0000000000049742

**Published:** 2026-07-10

**Authors:** Lin Chen, Wei Zheng, LinHua Liu

**Affiliations:** a Department of Endocrinology, Fuzhou University Affiliated Provincial Hospital, Fuzhou, China; b Department of Endocrinology, Shengli Clinical Medical College, Fujian Medical University, Fuzhou, China; c Department of Neurology, Fujian Provincial Geriatric Hospital, Fuzhou, China.

**Keywords:** cardiovascular-kidney-metabolic syndrome, disease progression, frailty, growth mixture modeling, trajectory

## Abstract

The impact of dynamic cardiovascular-kidney-metabolic (CKM) syndrome progression on longitudinal frailty trajectories remains unclear. This study investigated how rapid CKM worsening influences adverse frailty index development. We analyzed 3090 community-dwelling adults (baseline CKM stages 1–3, non-frail). CKM progression over 4 years was categorized as stable/improved, mild (+1 stage), or rapid (≥2 stages). Growth mixture modeling identified latent frailty index trajectories over a 7-year follow-up. Growth mixture modeling identified 2 trajectories: “Stable Progressive” (93.5%) and “Accelerated Adverse” (6.5%). Multivariable analysis revealed that rapid CKM progression significantly increased the risk of the “Accelerated Adverse” trajectory (OR = 2.44; 95% CI: 1.56–3.83) compared to the stable/improved group. Notably, mild progression showed no significant association. Rapid CKM stage deterioration independently predicts accelerated frailty worsening. Incorporating dynamic CKM changes into clinical risk assessments is crucial for preventing the transition from cardiometabolic multimorbidity to severe frailty.

## 1. Introduction

As population aging accelerates globally, the medical community confronts the intersection of 2 critical health challenges: the newly defined Cardiovascular-Kidney-Metabolic (CKM) syndrome and the geriatric syndrome of frailty. The American Heart Association’s (AHA) 2023 framework redefines cardiovascular risk not as isolated organ failure, but as a systemic entanglement of metabolic dysregulation, chronic kidney disease (CKD), and cardiovascular pathology.^[1]^ This multisystem dysfunction is inextricably linked to frailty – a state of diminished physiological reserve that precipitates disability and mortality.^[2,3]^ While advanced CKM stages are known predictors of cardiovascular death and shortened life expectancy, the impact of CKM on functional aging is equally profound.^[4]^ Understanding how this complex multimorbidity accelerates the transition from robustness to frailty is critical for developing integrated prevention strategies that preserve independence in older adults.

Existing literature has established a robust, graded association between CKM burden and adverse outcomes. Cross-sectional and longitudinal studies consistently show that higher baseline CKM stages correlate with increased frailty indices and mortality.^[5,6]^ Similarly, research in metabolic syndrome and CKD has demonstrated that cumulative physiological dysregulation acts as a driver of functional decline and frailty progression.^[4,7]^ However, the current understanding remains constrained by a static perspective. Most studies rely on single-point baseline assessments, asking only whether current disease severity predicts future frailty or mortality.^[6]^ This approach overlooks the dynamic nature of the syndrome: clinical reality demonstrates that patients with identical baseline stages may follow drastically different trajectories – some remain stable, while others experience a rapid, multi-stage deterioration over a short period.^[7,8]^

Despite the recognition of CKM as a progressive disorder, critical knowledge gaps persist. First, few studies have quantified the velocity of CKM progression (e.g., rapid vs mild worsening) as an independent exposure or examined whether progression between stages carries gradient risk. It remains unclear whether the rate of change exerts a pathogenic effect distinct from the state of the disease itself. Second, the outcome of frailty is often oversimplified as a linear average change or a binary status, masking the heterogeneity of aging.^[9]^ Previous models have rarely identified latent subgroups of older adults who, driven by systemic CKM collapse or metabolic burden, fall into an “accelerated adverse” frailty trajectory – a phenotype likely requiring urgent intervention.^[10]^

This longitudinal cohort study examines how changes in CKM progression affect long-term frailty patterns in middle-aged and older adults living in the community. Leveraging the 2023 AHA framework, we move beyond static baselines to classify CKM dynamics over a 4-year interval into distinct phenotypes: stable/improved, mild progression (+1 stage), and rapid progression (≥2 stages).^[11]^ We employ growth mixture modeling (GMM) to identify latent classes of frailty development over a 7-year follow-up, an approach that captures heterogeneous frailty paths beyond average slopes.^[12]^ Our primary objective is to determine whether rapid CKM progression specifically predicts membership in an accelerated frailty trajectory, independent of baseline severity and traditional risk factors.

By explicitly linking the speed of CKM worsening to heterogeneous frailty patterns, this study aims to shift the paradigm from static risk stratification to dynamic monitoring. Identifying “rapid progressors” early could delineate a critical window of opportunity for multisystem interventions, potentially decoupling cardiometabolic disease from severe functional decline and informing more precise CKM–frailty–focused care pathways.

## 2. Study population

### 2.1. Data source

This longitudinal cohort study used data from the China Health and Retirement Longitudinal Study (CHARLS), a continuous, nationally representative survey aimed at monitoring the aging process in Chinese adults aged 45 and above.^[13]^ Previous publications have provided comprehensive descriptions of the study design and sampling techniques.^[14]^ The Biomedical Ethics Review Committee of Peking University approved the CHARLS protocol (Approval No. IRB00001052-11015), and all participants provided written informed consent.^[14]^

### 2.2. Study participants and selection criteria

The process for selecting participants is shown in Figure S1, Supplemental Digital Content 1. From the initial 17,708 respondents in the 2011 baseline survey, we applied sequential exclusions to define an at-risk cohort with complete longitudinal data. First, to minimize reverse causality and focus on metabolic risk progression, we excluded individuals with missing baseline CKM data (n = 3483), those at Stage 0 (no risk factors, n = 591), and those with prevalent clinical CVD (Stage 4, n = 2498). Second, to assess incident adverse frailty, we removed participants with missing baseline frailty data (n = 3020) or prevalent frailty [Frailty Index (FI) ≥ 0.25, n = 617]. Finally, to ensure robust trajectory modeling, we excluded participants missing exposure data in 2015 (n = 1758), incomplete outcome assessments during follow-up (2013–2018, n = 2577), or those aged < 45 years (n = 74). The final analytic sample comprised 3090 participants. Missing data, including BMI (6.34%), residence (0.03%), and alcohol status (0.03%), were multiple imputed by Chained Equations to generate 50 complete datasets.

### 2.3. Definition of CKM stages

Participants were stratified into 5 stages based on the 2023 AHA framework,^[1]^ Stage 0 (No Risk); Stage 1 (Excess Adiposity), defined by overweight/obesity or impaired glucose tolerance; Stage 2 (Metabolic Risk), including hypertension, diabetes, metabolic syndrome, or moderate CKD; Stage 3 (Subclinical CVD), defined by severe renal dysfunction (eGFR < 30 or macroalbuminuria) or high 10-year CVD risk (Framingham score ≥ 20%); and Stage 4 (Clinical CVD).^[15]^ CKM stage change from 2011 to 2015 was calculated as the difference between 2015 and 2011 stages and categorized into 3 groups: stable/improved (difference ≤ 0), mild progression (+1 stage), and rapid progression (≥+2 stages), treated as an ordinal variable (coded 1–3) with the stable/improved group as reference.

### 2.4. Assessment of frailty

The FI was developed using the conventional model of accumulating deficits.^[7]^ We incorporated health-related deficits available in the CHARLS, encompassing multiple domains including chronic comorbidities, functional limitations (activities of daily living and instrumental activities of daily living), cognitive impairment, and depressive symptoms. Each item was mapped to a score ranging from 0 (absence of deficit) to 1 (presence of deficit). The FI was calculated as the ratio of the accumulated deficit score to the total number of non-missing items for each participant and then multiplied by 100, yielding a continuous value between 0 and 100. Consistent with established epidemiological criteria,^[7,16]^ frailty was defined as an FI ≥ 25, indicating a state of significant physiological vulnerability.

### 2.5. Covariates

Referring to previous population-based studies,^[17,18]^ we accounted for a wide range of initial covariates gathered from structured interviews and physical exams. Age, gender, level of education, marital status, and place of residence were among the sociodemographic factors. According to prior literature,^[19,20]^ lifestyle behaviors comprised smoking and alcohol status (never, former, or current). BMI and a comorbidity profile, which included self-reported physician diagnoses of arthritis, chronic lung disease, and cancer, were used to adjust health status.

### 2.6. Statistical analysis

**Figure 1.**
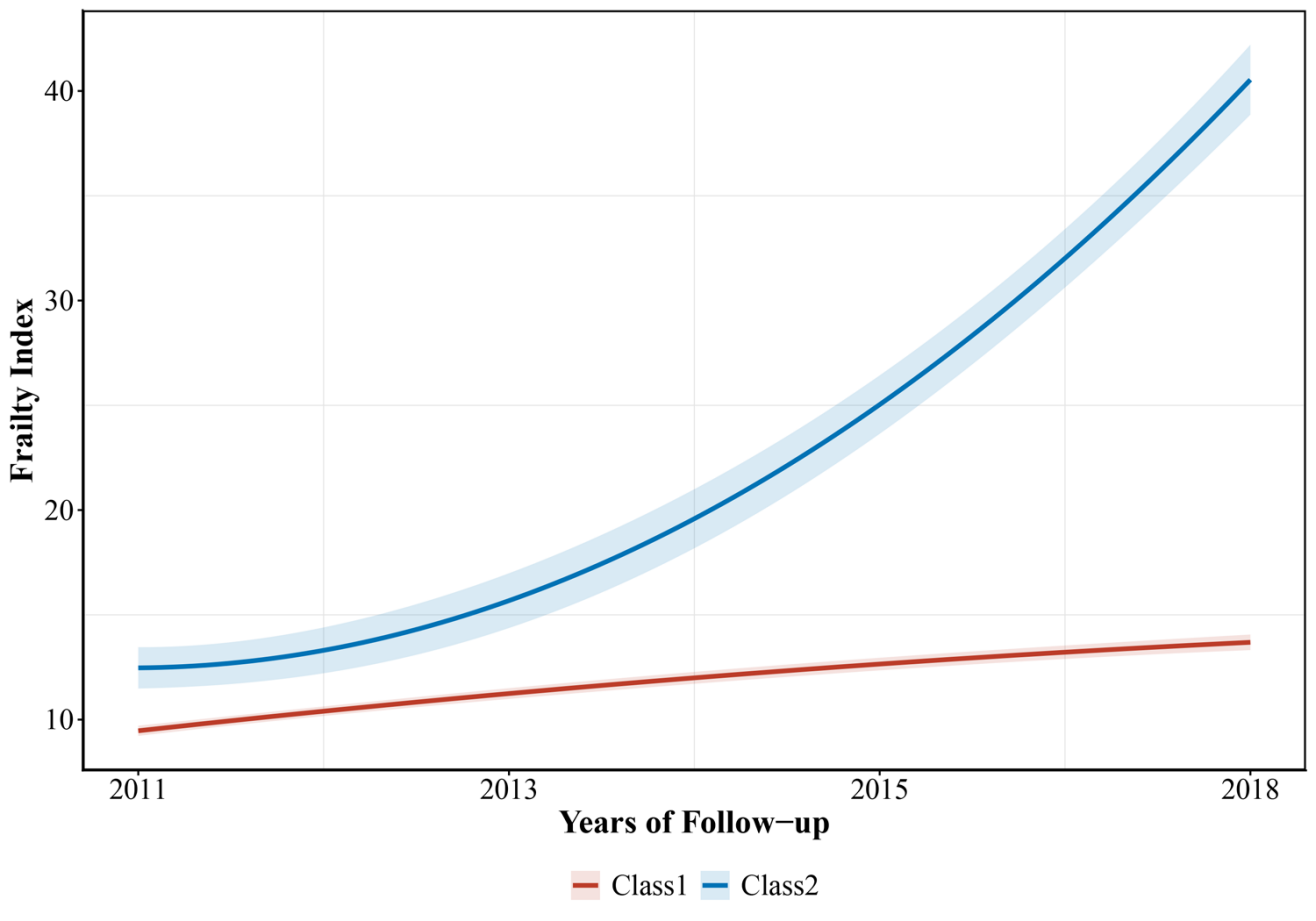
Trajectory groups of the FI (0–100 scale) from 2011 to 2018. FI = frailty index.

All analyses utilized data from the 2011 to 2018 waves of CHARLS, with 2011 serving as the baseline for covariate assessment. The primary outcome, longitudinal frailty trajectories, was identified using GMM based on FI measurements across 4 time points (2011, 2013, 2015, 2018).^[21]^ We assessed models ranging from 2 to 5 latent classes, determining the best fit using the Bayesian Information Criterion, Akaike Information Criterion, entropy, and clinical interpretability.^[21,22]^ Models with trajectory groups ranging from 2 to 5 were fitted to find the optimal model (Table S1, Supplemental Digital Content 2). This process yielded a binary outcome classification: “Stable Progressive” versus “Accelerated Adverse” trajectories (Fig. 1).^[23]^

Baseline characteristics were divided by trajectory group and analyzed using Student’s *t* tests, Mann–Whitney *U* tests, Chi-square tests, or Fisher exact tests, depending on suitability. Linear and logistic regression models were used for missing data imputation, with the final imputed values obtained through nearest neighbor matching within the predictive mean matching framework.

We employed multivariable logistic regression to examine the association between CKM stage progression (exposure) and Accelerated Adverse trajectory membership (outcome). Referring to previous large cohort studies,^[24,25]^ odds ratios along with 95% confidence intervals were estimated across 3 hierarchical models: Model 1 adjusted for baseline CKM stage to isolate the effect of progression velocity; Model 2 added sociodemographic factors (age, sex, education, marital status, residence); and Model 3 further adjusted for lifestyle behaviors, BMI, and comorbidities (smoking, alcohol, chronic lung disease, arthritis, cancer).

Subgroup analyses were stratified by age (<65 vs ≥65 years) and sex, with interaction terms tested for effect modification. Robustness was verified through 3 sensitivity analyses: modeling CKM stage change as a continuous variable using restricted cubic splines to test for nonlinearity; performing a complete-case analysis to validate imputation results; and calculating E-values to assess potential unmeasured confounding. The analyses were performed using R software (version 4.5.2) and Zstats v1.0, with statistical significance determined by a 2-sided *P*-value of <.05.

## 3. Results

### 3.1. Identification of FI trajectories

Latent GMM identified 2 distinct frailty trajectories from 2011 to 2018 (Fig. 1). Class 1 (“Stable Progressive,” 93.5%) exhibited a low baseline FI with gradual linear growth. Class 2 (“Accelerated Adverse,” 6.5%) featured a higher baseline FI and rapid, nonlinear acceleration, particularly in later follow-up.

### 3.2. Baseline characteristics

Among 3090 participants (mean age 56.7 years; 52.5% male), 73.4% remained in the CKM Stable group, while 18.2% and 8.4% experienced Mild and Rapid Progression, respectively. Compared to the Stable Progressive Trajectory group, the Accelerated Adverse group was notably older, predominantly female, had lower education levels, and exhibited higher incidences of chronic lung disease and arthritis (all *P* < .01). Notably, the prevalence of CKM Rapid Progression was nearly double in the Accelerated Adverse group (15.4% vs 7.9%; *P* < .001) (Table 1).

**Table 1:** Baseline characteristics of the study population according to frailty trajectory groups (N = 3090).

Characteristic	Overall (n = 3090)	Stable progressive (n = 2888)	Accelerated adverse (n = 202)	*P*-value
Age (years), mean, SD	56.70 ± 7.78	56.58 ± 7.77	58.45 ± 7.74	**.001**
Age group				
45–60 yr	2579 (83.46)	2424 (83.93)	155 (76.73)	**.008**
≥60 yr	511 (16.54)	464 (16.07)	47 (23.27)	
Sex, n (%)				
Male	1621 (52.46)	1537 (53.22)	84 (41.58)	**.001**
Female	1469 (47.54)	1351 (46.78)	118 (58.42)	
Smoking status, n (%)				
Former smoker	278 (9.00)	259 (8.97)	19 (9.41)	.449
Never smoker	1816 (58.77)	1690 (58.52)	126 (62.38)	
Current smoker	996 (32.23)	939 (32.51)	57 (28.22)	
Alcohol status, n (%)				
Former Drinker	210 (6.80)	193 (6.68)	17 (8.42)	**.002**
Never Drinker	1699 (54.98)	1568 (54.29)	131 (64.85)	
Current Drinker	1181 (38.22)	1127 (39.02)	54 (26.73)	
Educational level, n (%)				
High school or above	43 (1.39)	42 (1.45)	1 (0.50)	**<.001**
Less than primary education	1817 (58.80)	1664 (57.62)	153 (75.74)	
Primary school	815 (26.38)	779 (26.97)	36 (17.82)	
Middle school	415 (13.43)	403 (13.95)	12 (5.94)	
Residence, n (%)				
Urban	672 (21.75)	632 (21.88)	40 (19.80)	.488
Rural	2418 (78.25)	2256 (78.12)	162 (80.20)	
BMI (kg/m^2^, IQR)	24.21 ± 3.49	24.19 ± 3.47	24.52 ± 3.84	.196
Marital status, n (%)				
Unmarried	337 (10.91)	310 (10.73)	27 (13.37)	.246
Married	2753 (89.09)	2578 (89.27)	175 (86.63)	
Hypertension, n (%)	833 (26.96)	769 (26.63)	64 (31.68)	.117
Diabetes, n (%)	218 (7.06)	198 (6.86)	20 (9.90)	.102
Cancer, n (%)	13 (0.42)	12 (0.42)	1 (0.50)	.585
Chronic lung disease, n (%)	211 (6.83)	188 (6.51)	23 (11.39)	**.008**
Arthritis, n (%)	809 (26.18)	740 (25.62)	69 (34.16)	**.008**
Dyslipidemia, n (%)	319 (10.32)	301 (10.42)	18 (8.91)	.495
CKM syndrome, n (%)				
Stage 1	373 (12.07)	352 (12.19)	21 (10.40)	.502
Stage 2	2027 (65.60)	1897 (65.69)	130 (64.36)	
Stage 3	690 (22.33)	639 (22.13)	51 (25.25)	
CKM stage progression, n (%)				
CKM stable group	2268 (73.40)	2136 (73.96)	132 (65.35)	**<.001**
CKM mild progression group	562 (18.19)	523 (18.11)	39 (19.31)	
CKM rapid progression group	260 (8.41)	229 (7.93)	31 (15.35)	

The bold values represent those with P < 0.05, which we have used to denote statistical significance.

BMI = body mass index, CKM = cardiovascular-kidney-metabolic.

### 3.3. Association between CKM progression and FI trajectories

In the fully adjusted model, CKM Rapid Progression (≥2 stages) was independently associated with a 2.44-fold increased risk of membership in the Accelerated Adverse Trajectory (OR, 2.44; 95% CI, 1.56–3.83; *P* < .001). CKM Mild Progression (+1 stage) indicated a tendency for heightened risk (OR, 1.47; 95% CI, 0.97–2.22) but was not statistically significant (*P* = .069) (Table 2).

**Table 2:** Multivariable logistic regression analyses of the association between CKM stage progression and accelerated adverse frailty trajectory.

Variables	Model1		Model2		Model3	
	OR (95%CI)	P-value	OR (95%CI)	P-value	OR (95%CI)	P-value
CKM stage progression	–	–	–	–	–	–
CKM stable group	1.00 (Ref)	–	1.00 (Ref)	–	1.00 (Ref)	–
CKM mild progression group	1.37 (0.93–2.03)	0.111	1.27 (0.87–1.84)	0.216	1.47 (0.97–2.22)	0.069
CKM rapid progression group	2.51 (1.63–3.87)	**<.001**	2.19 (1.44–3.33)	**<.001**	2.44 (1.56–3.83)	**<.001**

Calculated using multivariable logistic regression with sequential adjustment across 3 models: Model 1 adjusted for CKM syndrome only; Model 2 additionally adjusted for demographic factors (age, sex, education level, marital status, and residence); Model 3 further adjusted for lifestyle, comorbidities, and body mass index (including alcohol status, smoking status, arthritis, chronic lung disease, and cancer).

The bold values represent those with P < 0.05, which we have used to denote statistical significance.

CI = confidence interval, CKM = cardiovascular-kidney-metabolic, OR = odds ratio.

### 3.4. Subgroup and sensitivity analyses

**Figure 2.**
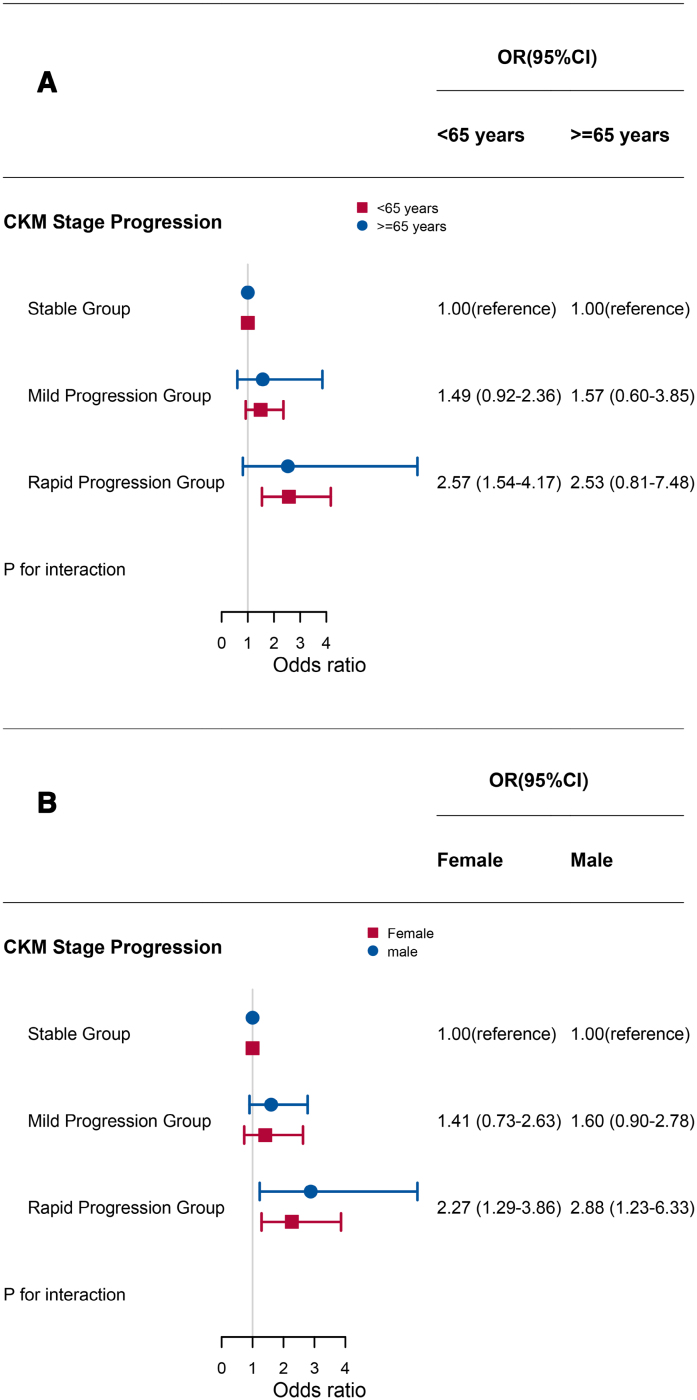
Subgroup analyses of the association between CKM stage progression and accelerated adverse frailty trajectory, stratified by age and sex. CKM = cardiovascular-kidney-metabolic.

**Figure 3.**
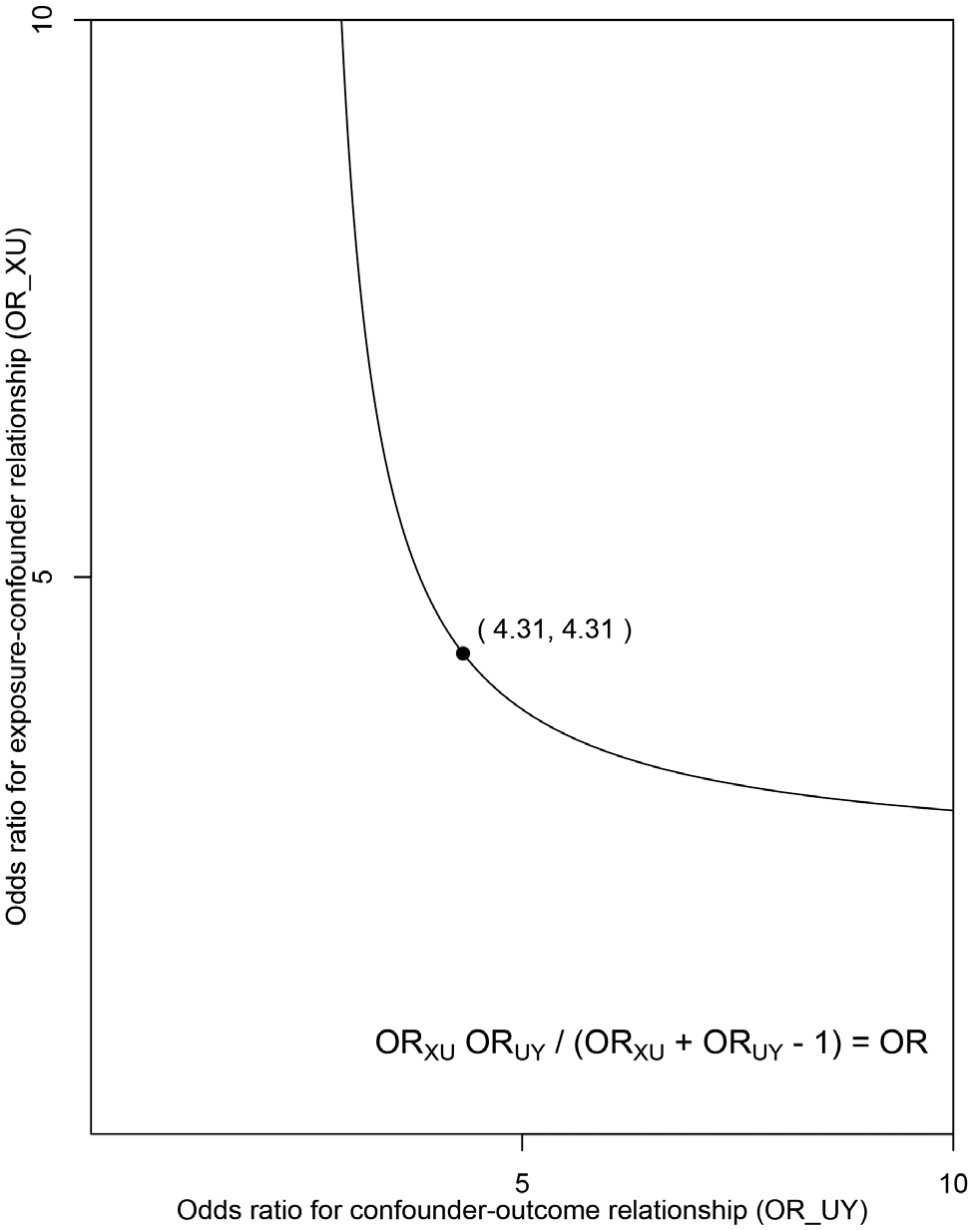
E-value sensitivity analysis for unmeasured confounding.

Subgroup analyses demonstrated consistent associations across age and sex strata, with no significant interactions observed (Fig. 2). Robustness was further substantiated through sensitivity analyses: continuous modeling of CKM stage change revealed a linear dose–response relationship (OR per unit increase, 1.39; 95% CI, 1.16–1.68), validated by restricted cubic splines (*P* for nonlinearity = .258; Table 3, Figure S2, Supplemental Digital Content 3); complete-case analysis yielded comparable estimates (OR, 2.51; 95% CI, 1.57–4.01; Table 4); and an E-value of 4.31 indicated substantial robustness to potential unmeasured confounding (Fig. 3).

**Table 3:** Sensitivity analysis using CKM stage change as a continuous variable.

Variables	Model 3	
	OR (95%CI)	*P*
CKM change	1.39 (1.16–1.68)	<.001

Model 3 adjusted for CKM syndrome, age, sex, education level, marital status, residence, alcohol status, smoking status, body mass index, arthritis, chronic lung disease, and cancer.

CI = confidence interval, OR = odds ratio.

**Table 4:** Sensitivity analysis using complete cases without imputation.

Variables	Model3	
	OR (95% CI)	*P*
CKM change group factor		
CKM stable group	1.00 (Reference)	–
CKM mild progression group	1.38 (0.89–2.15)	0.146
CKM rapid progression group	2.51 (1.57–4.01)	<.001

Model 3 adjusted for CKM syndrome, age, sex, education level, marital status, residence, alcohol status, smoking status, body mass index, arthritis, chronic lung disease, and cancer.

CI = confidence interval, CKM = cardiovascular-kidney-metabolic, OR = odds ratio.

## 4. Discussion

This longitudinal cohort study suggests that the velocity of CKM syndrome progression may be associated with distinct frailty trajectories in older adults. By characterizing dynamic disease transitions over a 4-year period, we demonstrated that rapid progression of CKM syndrome – defined as an escalation of 2 or more stages – acts as a potent and independent driver of an accelerated adverse frailty trajectory. Conversely, mild progression showed a nonsignificant trend toward deterioration, which may suggest a possible threshold effect whereby physiological resilience buffers gradual insults but may become less effective in the setting of rapid multisystem decompensation.

These findings suggest that dynamic monitoring of disease velocity may complement static disease burden assessment and help identify individuals at higher risk of functional decline.^[2,7]^

Our primary finding – that the rate of CKM worsening is a stronger predictor of adverse outcomes than static baseline measures – aligns with and extends the emerging “dynamic risk” paradigm in chronic disease epidemiology. Prior studies with a cross-sectional design have firmly established the gradual relationship between advanced CKM stages and frailty.^[5,26]^ However, these snapshot assessments inherently fail to capture the heterogeneity of aging. Our results mirror recent observations in diabetic kidney disease and multimorbidity, where the slope of biomarker deterioration often predicts mortality more accurately than absolute values.^[27]^ Uniquely, our study elucidates that this “velocity effect” extends beyond organ-specific failure to systemic functional collapse. By utilizing GMM, we uncovered that a minority subgroup (6.5%) experiences a catastrophic trajectory of frailty accumulation, a phenomenon that would likely be masked by traditional linear regression models that average population trends.^[28,29]^

A particularly intriguing finding is the distinct divergence in risk between mild (+1 stage) and rapid (≥2 stages) progressors. While mild progression was associated with a modest, nonsignificant increase in frailty risk, rapid progression conferred a nearly 2.5-fold excess risk.^[30]^ This nonlinearity may be consistent with a potential “tipping point” mechanism related to the concept of physiological reserve.^[31]^ It is plausible that gradual metabolic dysregulation allows for homeostatic adaptation – a state of “allostatic load” where functional capacity is maintained at a cost but without overt failure.^[32]^ However, rapid progression likely represents an acute, overwhelming assault on multiple organ systems simultaneously (e.g., simultaneous worsening of renal function and onset of hypertension). In this context, rapid multisystem deterioration may contribute to abrupt depletion of physiological reserves, potentially overwhelming compensatory mechanisms and accelerating processes such as sarcopenia, neuroendocrine dysregulation, and chronic inflammation that have been linked to the frailty phenotype.^[33,34]^ Furthermore, rapid CKM progression may reflect, rather than directly demonstrate, biological processes such as a heightened inflammatory burden or mitochondrial dysfunction, which have been proposed as shared pathological pathways underlying both cardiovascular aging and physical frailty.^[35]^

The identification of “rapid progressors” as a distinct high-risk phenotype has immediate clinical implications. Our data argue for the integration of “trajectory monitoring” into primary care. A patient who transitions from CKM Stage 1 to Stage 3 within a short interval (e.g., 4 years) should be flagged as a “Red Flag” case, warranting comprehensive geriatric assessment and aggressive multisystem intervention, even if their functional status appears preserved at the moment.^[36]^ This aligns with the AHA’s call for holistic care models but adds a temporal dimension: the speed of disease evolution is a vital sign.^[37]^ Intervening during the phase of rapid CKM progression may represent a “window of opportunity” to decouple metabolic disease from irreversible disability.

The study’s advantages include its prospective nature, the rigorous exclusion of prevalent frailty to minimize reverse causality, and the application of GMM to capture heterogeneous aging trajectories. Our findings are further validated by E-value analyses, which suggest minimal bias from unmeasured confounding.

However, limitations warrant consideration. First, although key metabolic biomarkers were measured objectively, data on specific comorbidities and lifestyle factors depended on self-reports, which might introduce recall bias. Second, despite the use of multiple imputation, possible selection bias resulting from participant attrition or the “healthy survivor effect” cannot be entirely ruled out in this older cohort. Finally, as the study population is exclusively Chinese, generalizability to other ethnicities with different metabolic profiles requires verification.

## 5. Conclusion

Rapid progression of CKM syndrome serves as a potent, independent predictor of accelerated frailty, suggesting a critical threshold of physiological resilience where multisystem collapse precipitates functional decline. These findings advocate for a paradigm shift from static risk assessment to dynamic trajectory monitoring. Future research should elucidate the underlying molecular mechanisms and evaluate whether aggressive CKM-targeted interventions can mitigate adverse frailty trajectories.

## Author contributions

**Conceptualization:** Lin Chen, Wei Zheng.

**Data curation:** Lin Chen, Wei Zheng.

**Formal analysis:** Lin Chen, Wei Zheng.

**Funding acquisition:** Lin Chen.

**Investigation:** LinHua Liu.

**Methodology:** Lin Chen.

**Project administration:** LinHua Liu.

**Resources:** LinHua Liu.

**Supervision:** LinHua Liu.

**Validation:** LinHua Liu.

**Writing – original draft:** Lin Chen, Wei Zheng.

**Writing – review & editing:** Lin Chen.







## References

[1] Ndumele CE Rangaswami J Chow SL; American Heart Association. Cardiovascular-kidney-metabolic health: a presidential advisory from the American Heart Association. Circulation. 2023;148:1606–35. 37807924 10.1161/CIR.0000000000001184

[2] Zhang C Hao C Liang W. Abdominal obesity and frailty progression in population across different cardiovascular-kidney-metabolic syndrome stages: a nationwide longitudinal study. Diabetol Metab Syndr. 2025;17:75. 40033331 10.1186/s13098-025-01649-0 PMC11877786

[3] Kaskirbayeva D West R Jaafari H. Progression of frailty as measured by a cumulative deficit index: a systematic review. Ageing Res Rev. 2023;84:101789. 36396032 10.1016/j.arr.2022.101789

[4] Claudel SE Schmidt IM Waikar SS Verma A. Cumulative incidence of mortality associated with cardiovascular-kidney-metabolic (CKM) syndrome. J Am Soc Nephrol. 2025;36:1343–51. 39932805 10.1681/ASN.0000000637 PMC12187233

[5] Li X Zhao W Zhao L Sun T Zhu F Wang D. Association between the frailty and cardiovascular disease risk in populations with cardiovascular-kidney-metabolic syndrome stages 0-3: a prospective cohort study [published online ahead of print October 7, 2025]. Am J Nephrol. doi: 10.1159/000548772. 10.1159/000548772 41056203

[6] Li J Wei X. Association of cardiovascular-kidney-metabolic syndrome with all-cause and cardiovascular mortality: a prospective cohort study. Am J Prev Cardiol. 2025;22:100985. 40242364 10.1016/j.ajpc.2025.100985 PMC12003006

[7] Zeng P Li M Cao J Zeng L Jiang C Lin F. Association of metabolic syndrome severity with frailty progression among Chinese middle and old-aged adults: a longitudinal study. Cardiovasc Diabetol. 2024;23:302. 39152431 10.1186/s12933-024-02379-9 PMC11329990

[8] Jang AR Sagong H Yoon JY. Frailty trajectory among community-dwelling middle-aged and older adults in Korea: evidence from the Korean longitudinal study of aging. BMC Geriatr. 2022;22:524. 35752752 10.1186/s12877-022-03229-7 PMC9233334

[9] Verghese J Ayers E Sathyan S. Trajectories of frailty in aging: prospective cohort study. PLoS One. 2021;16:e0253976. 34252094 10.1371/journal.pone.0253976 PMC8274857

[10] Du J Ye F Zhang M. Development and validation of nomograms to predict frailty-worsening trajectories among Chinese older adults. Front Public Health. 2025;13:1588303. 40746700 10.3389/fpubh.2025.1588303 PMC12312636

[11] Zhu Y Wang X Wang K. Association between baseline cardio-kidney-metabolic syndrome, its transition and cognitive impairment: result from CHARLS study. Diabetol Metab Syndr. 2025;17:211. 40514737 10.1186/s13098-025-01779-5 PMC12164072

[12] Muthén B Muthén LK. Integrating person-centered and variable-centered analyses: growth mixture modeling with latent trajectory classes. Alcohol Clin Exp Res. 2000;24:882–91. 10888079

[13] Luo YX Zhou XH Heng T. Bidirectional transitions of sarcopenia states in older adults: the longitudinal evidence from CHARLS. J Cachexia Sarcopenia Muscle. 2024;15:1915–29. 39001569 10.1002/jcsm.13541 PMC11446714

[14] Zhao Y Hu Y Smith JP Strauss J Yang G. Cohort profile: the China health and retirement longitudinal study (CHARLS). Int J Epidemiol. 2014;43:61–8. 23243115 10.1093/ije/dys203 PMC3937970

[15] Hu Y Liang Y Li J Li X Yu M Cui W. Correlation between atherogenic index of plasma and cardiovascular disease risk across cardiovascular-kidney-metabolic syndrome stages 0-3: a nationwide prospective cohort study. Cardiovasc Diabetol. 2025;24:40. 39856691 10.1186/s12933-025-02593-z PMC11763136

[16] He D Wang Z Li J. Changes in frailty and incident cardiovascular disease in three prospective cohorts. Eur Heart J. 2024;45:1058–68. 38241094 10.1093/eurheartj/ehad885

[17] You Y Cui Y Zheng K. Oral microbiome diversity shapes the association between leisure-time physical activity and cognitive function among older adults. iScience. 2026;29:115086. 41847618 10.1016/j.isci.2026.115086 PMC12989845

[18] YouYLiJZhangY. Exploring the role of β2-microglobulin in the relationship between physical activity and DNAm-predicted PhenoAge: evidence from a population-based and mice single-cell RNA-sequencing study [published online ahead of print November 22, 2025]. J Adv Res. doi: 10.1016/j.jare.2025.11.047.10.1016/j.jare.2025.11.04741285310

[19] You Y Ding H Tang M. Dose-response relationship between leisure-time physical activity and metabolic syndrome in short sleep US adults: evidence from a nationwide investigation. Appl Physiol Nutr Metab. 2025;50:1–10. 10.1139/apnm-2024-0347 39993280

[20] You Y Zheng K Ablitip A. Life’s essential 8 and depression: a national cross-sectional analysis in US emerging adults. J Adolesc Health. 2025;77:84–93. 40445158 10.1016/j.jadohealth.2025.03.023

[21] Nguena Nguefack HL Pagé MG Katz J. Trajectory modelling techniques useful to epidemiological research: a comparative narrative review of approaches. Clin Epidemiol. 2020;12:1205–22. 33154677 10.2147/CLEP.S265287 PMC7608582

[22] van der Nest G Lima Passos V Candel M van Breukelen GJP. An overview of mixture modelling for latent evolutions in longitudinal data: modelling approaches, fit statistics and software. Adv Life Course Res. 2020;43:100323. 36726256 10.1016/j.alcr.2019.100323

[23] Kwon JY Sawatzky R Baumbusch J Lauck S Ratner PA. Growth mixture models: a case example of the longitudinal analysis of patient-reported outcomes data captured by a clinical registry. BMC Med Res Methodol. 2021;21:79. 33882863 10.1186/s12874-021-01276-z PMC8058975

[24] You Y Wang D Ding H. Mediation role of telomere length in the relationship between physical activity and PhenoAge: a population-based study. J Exerc Sci Fit. 2025;23:149–56. 40235556 10.1016/j.jesf.2025.03.004 PMC11994304

[25] You Y Chen Y Chen X. Threshold effects of the relationship between physical exercise and cognitive function in the short-sleep elder population. Front Aging Neurosci. 2023;15:1214748. 37424629 10.3389/fnagi.2023.1214748 PMC10323428

[26] Khan SS Coresh J Pencina MJ; American Heart Association. Novel prediction equations for absolute risk assessment of total cardiovascular disease incorporating cardiovascular-kidney-metabolic health: a scientific statement from the American Heart Association. Circulation. 2023;148:1982–2004. 37947094 10.1161/CIR.0000000000001191

[27] Tian Y Wang J Zhu T. Biological age acceleration associated with the progression trajectory of cardio-renal-metabolic multimorbidity: a prospective cohort study. Nutrients. 2025;17:1783. 40507052 10.3390/nu17111783 PMC12157706

[28] Miao X Guo Y Chen Y. Exploration of frailty trajectories and their associations with health outcomes in older gastric cancer survivors undergoing radical gastrectomy: a prospective longitudinal observation study. Eur J Surg Oncol. 2024;50:107934. 38160495 10.1016/j.ejso.2023.107934

[29] Stow D Matthews FE Hanratty B. Frailty trajectories to identify end of life: a longitudinal population-based study. BMC Med. 2018;16:171. 30236103 10.1186/s12916-018-1148-x PMC6148780

[30] Wang Z Fang L Wang L Zhang J. Non-linear associations and threshold effects of oxidative balance score and composite dietary antioxidant index on frailty risk in patients with cardiovascular-kidney-metabolic syndrome. Front Nutr. 2025;12:1673736. 41256923 10.3389/fnut.2025.1673736 PMC12620448

[31] Fried LP Cohen AA Xue QL Walston J Bandeen-Roche K Varadhan R. The physical frailty syndrome as a transition from homeostatic symphony to cacophony. Nat Aging. 2021;1:36–46. 34476409 10.1038/s43587-020-00017-z PMC8409463

[32] Wilkinson TJ Miksza J Zaccardi F. Associations between frailty trajectories and cardiovascular, renal, and mortality outcomes in chronic kidney disease. J Cachexia Sarcopenia Muscle. 2022;13:2426–35. 35851589 10.1002/jcsm.13047 PMC9530530

[33] Mirkowski K Vellone E Żółkowska B. Frailty and heart failure: clinical insights, patient outcomes and future directions. Card Fail Rev. 2025;11:e05. 40083651 10.15420/cfr.2024.34 PMC11904417

[34] Ijaz N Buta B Xue QL. Interventions for frailty among older adults with cardiovascular disease: JACC state-of-the-art review. J Am Coll Cardiol. 2022;79:482–503. 35115105 10.1016/j.jacc.2021.11.029 PMC8852369

[35] Ferrucci L Fabbri E. Inflammageing: chronic inflammation in ageing, cardiovascular disease, and frailty. Nat Rev Cardiol. 2018;15:505–22. 30065258 10.1038/s41569-018-0064-2 PMC6146930

[36] Kim BS Kim HJ Kim H Lee J Shin JH Sung KC. Longitudinal changes in cardiovascular-kidney-metabolic syndrome stages and their impact on outcomes: a nationwide cohort study. J Clin Med. 2025;14:3888. 40507650 10.3390/jcm14113888 PMC12156345

[37] Goldfarb MJ Saylor MA Bozkurt B; American Heart Association Council on Clinical Cardiology; Council on Cardiovascular and Stroke Nursing; Council on Hypertension; Council on Lifestyle and Cardiometabolic Health; Council on Peripheral Vascular Disease; and Council on Quality of Care and Outcomes Research. Patient-centered adult cardiovascular care: a scientific statement from the American Heart Association. Circulation. 2024;149:e1176–88. 38602110 10.1161/CIR.0000000000001233

