# Supplementary figures and images for: Rapid progression of cardiovascular-kidney-metabolic syndrome drives accelerated frailty trajectories: A longitudinal cohort study
Source: Medicine (Baltimore). 2026 Jul 10;105(28):e49742. doi: 10.1097/MD.0000000000049742 (PMC13362927; doi:10.1097/MD.0000000000049742)

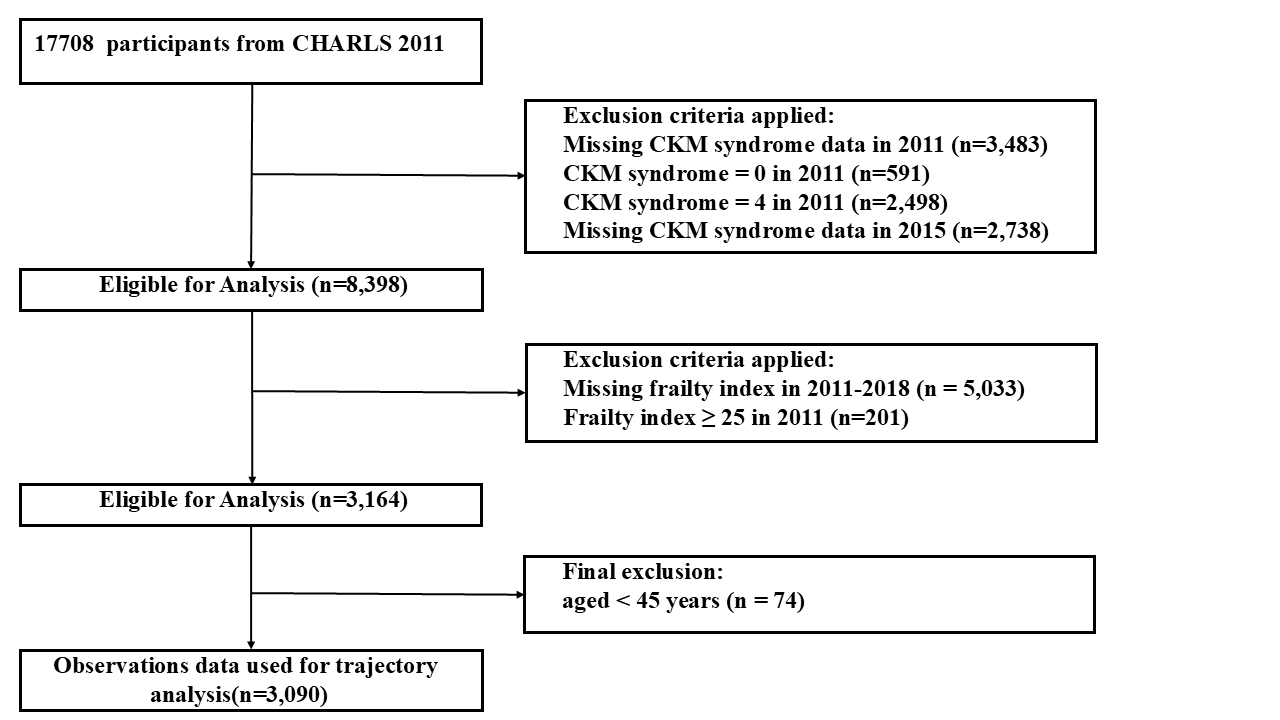

Supplement: Supplementary file 1 [file medi-105-e49742-s001.tif]

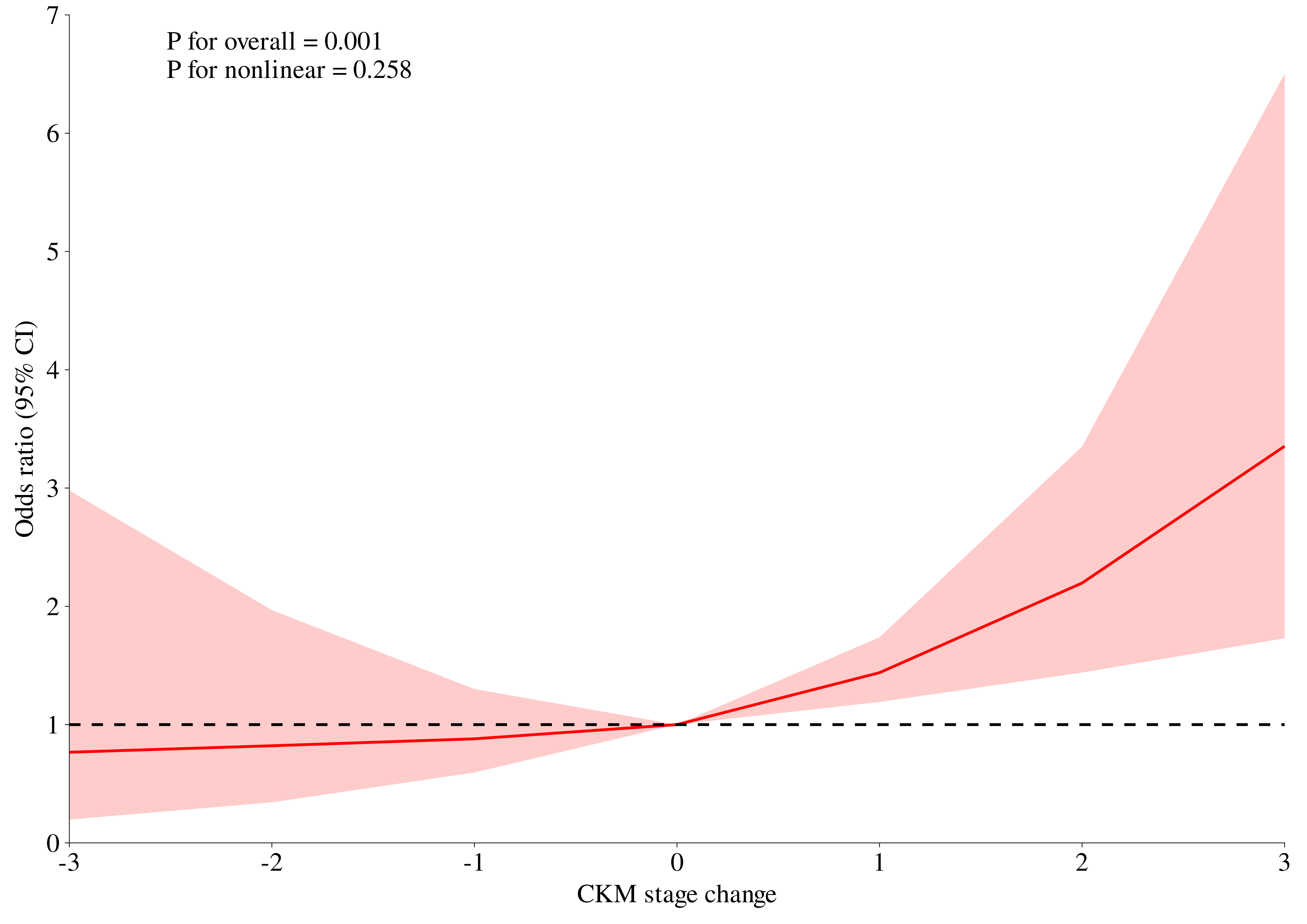

Supplement: Supplementary file 3 [file medi-105-e49742-s003.tif]
