# Supplementary material for: Rapid progression of cardiovascular-kidney-metabolic syndrome drives accelerated frailty trajectories: A longitudinal cohort study
Source: Medicine (Baltimore). 2026 Jul 10;105(28):e49742. doi: 10.1097/MD.0000000000049742 (PMC13362927; doi:10.1097/MD.0000000000049742)
Supplement: Supplementary file 2 [file medi-105-e49742-s002.docx]

**Table S1: Fit indices of GMM model on frailty index trajectories.**

| Model | BIC | aBIC | AIC | Entroy | Class (%) |
| --- | --- | --- | --- | --- | --- |
| 1 | **82346.799** | 82324.557 | 82304.548 | 1.00 | 100.0 |
| 2 | **81528.418** | 81493.466 | **81462.023** | **0.929** | 93.46 / 6.54 |
| 3 | **81525.000** | 80865.045 | **81455.000** | **0.785** | 91 / 5/ 4 |
| 4 | 80944.850 | 80884.479 | 80830.167 | 0.497 | 0 / 87.22 / 6.21 / 6.57 |
| 5 | 80978.073 | 80904.993 | 80839.247 | 0.643 | 88.16 / 0 / 0 / 5.92 /5.92 |

Abbreviations: BIC, Bayesian Information Criterion; aBIC, adjusted Bayesian Information Criterion; AIC, Akaike Information Criterion; GMM, growth mixture modeling.
